# Longitudinal study of wild koalas (*Phascolarctos cinereus*) reveals chlamydial disease progression in two thirds of infected animals

**DOI:** 10.1038/s41598-019-49382-9

**Published:** 2019-09-13

**Authors:** Amy Robbins, Jonathan Hanger, Martina Jelocnik, Bonnie L. Quigley, Peter Timms

**Affiliations:** 1 Genecology Research Centre, University of the Sunshine Coast, 90 Sippy Downs Drive, Sippy Downs, Queensland 4556 Australia; 2 Endeavour Veterinary Ecology Pty Ltd, 1695 Pumicestone Road, Toorbul, Queensland 4510 Australia

**Keywords:** Ecological epidemiology, Applied microbiology, Clinical microbiology, Pathogens

## Abstract

Chlamydial disease threatens many of Australia’s koala populations, and yet our understanding of chlamydial epidemiology and disease dynamics in koalas is limited by a lack of comprehensive, longitudinal population studies. To address this, we utilised longitudinal samples from a large-scale population study of wild koalas in south-east Queensland, to follow chlamydial infections over time and to investigate some of the drivers of disease progression. Our findings show, firstly, that almost two thirds of chlamydial infections progressed to disease, challenging the notion that chlamydial infections in koalas commonly remain chronic and asymptomatic. Secondly, disease progression at the urogenital tract site was associated with infection load, and urogenital tract shedding was significantly higher when koalas acquired a new infection. Thirdly, chronic chlamydial exposure was not necessary for pathogenic sequelae to develop, such as infertility and mortality. Fourthly, *omp*A-characterised strain sub-types may reflect tissue tropisms and pathogenicity, and the chlamydial status of some chronically infected koalas may be explained by reinfections with novel genotypes. Finally, successful antimicrobial treatment provided only short-term protection against reinfection and disease progression in susceptible koalas. These findings highlight the importance of identifying and preventing chlamydial infections in koalas, informing new population management strategies and research priorities.

## Introduction

Australia’s northern koala populations are being pushed towards extinction by ongoing habitat degradation due to urban and agricultural development^1^, and as they decline, the threat of chlamydial disease is likely to become more significant. Chlamydial disease has been shown to negatively impact the viability of declining koala populations due to effects on mortality and reproductive rates, with up to 57% of female koalas infertile in some populations^2^ and disease identified as the largest single contributor to mortality in others^3^. Chlamydial disease control has subsequently been identified as a potential population management tool^3^, and successful conservation outcomes have been demonstrated with this approach. For example, a reduction in the prevalence of chlamydial disease in a declining south-east Queensland (SE Qld) koala population, from 20% to 4%, contributed to positive population growth rates after management interventions^4^. Two species of *Chlamydia* infect koalas, *C. pneumoniae* and *C. pecorum*, but disease is predominantly associated with *C. pecorum*^5^. Chlamydial infections in koalas, as with other hosts, are often characterised by asymptomatic carriage^6^. However, in susceptible individuals, they can cause inflammatory and fibrotic disease in the urinary and reproductive tracts, at the ocular site^5^, and occasionally in the respiratory tract^7^. Attempts have been made to identify the factors that drive chlamydial disease progression in these individuals^8^, however, despite its importance, a comprehensive understanding of chlamydial epidemiology and disease dynamics in koalas is lacking.

Previously suggested drivers of chlamydial disease progression in koalas include several pathogen-associated factors, such as the chlamydial strain and infection load. Chlamydial disease prevalence and severity varies between koala populations, with a higher prevalence and severity reported in the northern part of Australia^5,9,10^. One hypothesis to explain this pattern of disease occurrence is the geographical distribution of genetically distinct chlamydial strains, as characterised by chlamydial outer membrane protein A (*omp*A) genotyping. Victorian koalas, for example, are largely infected with apparently less pathogenic strains, characterised as *omp*A genotype B^11^. In SE Qld koalas, however, strains with specific *omp*A genotypes have previously not been associated with a significant risk of disease^12^. The relationship between the chlamydial infection load and disease progression in koalas is also unclear, with a significant association reported in only one^13^ of two studies from the same region^12^. However, the former study included data from hospitalised koalas, which may have biased the findings. Although these pathogen-associated factors are likely to be important in chlamydial disease progression, our understanding of their role is clearly limited, and chlamydial pathogenesis may also be dependent on several host-associated factors.

Host-associated factors, such as host genetics, immune phenotype, coinfection with immunosuppressive pathogens (such as koala retrovirus, KoRV), and the physiological response to stress, have also been suggested as drivers of chlamydial disease progression in koalas. Host genetics are known to contribute to chlamydial pathogenesis, with three major histocompatibility complex class II (MHC class II) variants being correlated with chlamydial disease in koalas^14^. In addition, high antibody titres against chlamydial heat shock proteins, associated with chlamydial disease in other host species^15^, were identified in koalas with tubal infertility^16^. Links have also been established between KoRV-B and chlamydial disease in SE Qld koalas^17^ and KoRV-A and cystitis in Victorian koalas^18^. Alternatively, the complementary geographical distribution of KoRV infections may explain the variability in chlamydial disease prevalence and severity between northern and southern koala populations. The physiological response to chronic stressors might also influence susceptibility to chlamydial disease, due to effects on immune function^19,20^. However, while there was a higher prevalence of chlamydial infections in overabundant Victorian koalas residing in a highly disturbed environment, this was not true for chlamydial disease^9^. The interactions between these host and pathogen-associated drivers of chlamydial disease progression are clearly complex, and further research is needed to determine the relative contribution of each factor.

Much of our current understanding of chlamydial epidemiology and disease dynamics in koalas has been derived from opportunistic, cross-sectional field studies or hospital data sets. Historically, field studies are likely to have severely underestimated the prevalence of chlamydial disease in wild koala populations, limited by a lack of comprehensive clinical examinations that included ultrasonography^9,11^. Further, field studies do not always examine all chlamydial-affected anatomical sites or both sexes^9,11^, and because of their opportunistic or cross-sectional nature, do not provide comprehensive data sets on chlamydial epidemiology and disease dynamics over time. The sensitivity and discriminatory power of diagnostic capabilities for *Chlamydia* has also drastically improved with the recent development of species-specific quantitative polymerase chain reaction (qPCR) assays and *Chlamydia* genotyping methods. The historical reliance in chlamydial research on hospital data sets, due to the costs associated with large-scale population studies of wild koalas, has also confounded the data, as these are inherently biased towards koalas showing overt signs of disease or residing near human habitation or roads^6^. To develop an accurate and thorough understanding of chlamydial epidemiology and disease dynamics in koalas, comprehensive, longitudinal population studies are required.

To address some of these limitations, we utilised a unique, large-scale, longitudinal data set from SE Qld koalas that included comprehensive, standardised clinical examinations and molecular diagnostics to investigate some of the factors driving chlamydial disease progression. Our findings characterise infection and disease dynamics over time and challenge the current paradigm that a large proportion of chlamydial infections in koalas remain chronic and asymptomatic.

## Results

**Figure 1:**
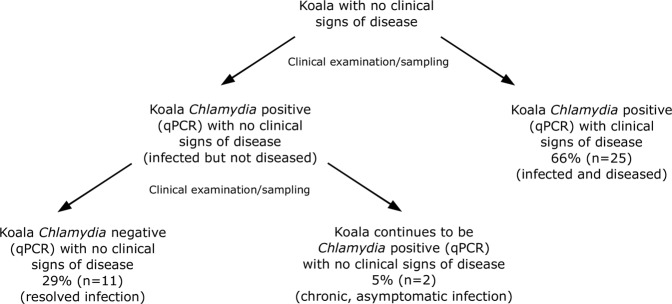
Proportion of new chlamydial infections in a population of south-east Queensland koalas that either progress to disease, are resolved by the host without progressing to disease, or remain chronic and asymptomatic.

We analysed comprehensive, longitudinal clinical records, compiled by koala-experienced veterinarians, and ocular conjunctiva and urogenital tract swab samples using *C. pecorum*-specific qPCR and *omp*A genotyping, to investigate some of the drivers of chlamydial disease progression and disease dynamics in a wild koala population in SE Qld (Fig. 1).

### Disease was the outcome for 2/3 of all chlamydial infections

From the longitudinal monitoring of 148 koalas over a total of 479 individual sampling points, 30 koalas met our study inclusion criteria by having a new chlamydial infection detected at the ocular or urogenital tract site by qPCR (Supplementary Table S1). These koalas had no evidence of chlamydial infection (qPCR below detection limit) or disease (clinical examination within normal limits) at their previous clinical examination. If disease was detected at their first clinical examination they were excluded from disease progression analyses only (unless it was their first sampling as an independent offspring, n = 1).

Overall, 66% (25/38) of these new chlamydial infections progressed to disease (Fig. 1). For 50% (19/38) of these infections, disease was also detected at the same time, at the same anatomical site. These koalas had no evidence of infection or disease at their previous clinical examination (1 to 9 months previously). For the remaining 16% (6/38) of these infections, the infection was present for one or more consecutive clinical examinations (2.5 to 18 months previously) before disease was detected at the same anatomical site. However, there was some overlap between the sampling schedules of infected koalas who were also diseased at their first infection time point and those who were chronically infected before they developed disease.

For 29% (11/38) of these new chlamydial infections, the koala was able to resolve the infection (as determined by the qPCR result becoming negative) at that anatomical site by the subsequent clinical examination (3.5 to 6 months later) without progressing to disease. Only 5% (2/38) of chlamydial infections were persistent infections at an anatomical site for more than one consecutive clinical examination, neither being resolved by the koala nor progressing to disease (Supplementary Fig. S1). These chronic, asymptomatic infections were followed for 22 months and 28 months until the conclusion of the koala management program, without progressing to disease or being resolved.

### The ability of a koala to resolve an infection was significantly associated with the anatomical site

Urogenital tract infections were significantly less likely to be resolved by the koala than ocular infections (13%; 4/31 vs 85%; 6/7) (two-tailed Fisher’s exact test, *p* < 0.001). Overall, 55% (6/11) of resolved infections occurred at the ocular site, 36% (4/11) of resolved infections occurred at the urogenital tract site and 9% (1/11) of resolved infections occurred at both sites (i.e. multifocal infections).

### Disease progression at the urogenital tract site was significantly associated with the infection load

**Figure 2:**
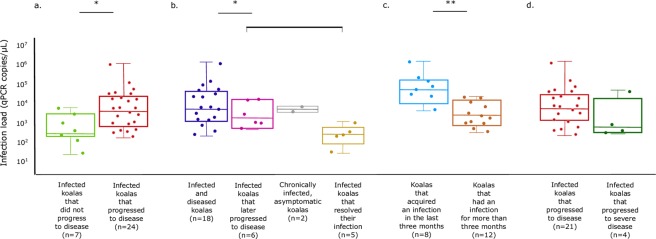
Comparisons of urogenital tract infection loads and disease dynamics in a population of south-east Queensland koalas. Statistical tests: (**a**,**c**,**d**) Mann Whitney U test, (**b**) Kruskal-Wallis test, *significance at *p* < 0.05, **significance at *p* < 0.001.

The median chlamydial infection load from swab samples collected at the urogenital tract site (qPCR copies/µL) was significantly higher when infections progressed to disease, compared to infections that were able to be resolved by the koala, or were chronic and asymptomatic (Mann-Whitney U = 34.0, *p* = 0.017) (Fig. 2a).

In addition, the chlamydial infection load at the urogenital tract site was significantly higher when infections were detected at the same clinical examination as disease, in comparison to infections that were present for one or more clinical examination before disease was detected or did not progress to disease (Kruskal-Wallis test, *p* = 0.019) (Fig. 2b).

### Urogenital tract infection loads declined in long-term infections

Urogenital tract infection loads were significantly higher when koalas acquired a new chlamydial infection, compared to koalas who had long-term infections (Mann-Whitney U = 4, *p* < 0.001) (Fig. 2c). Koalas were considered to have a new urogenital tract infection if they were *Chlamydia*-negative at their preceding clinical examination less than 3 months previously, while koalas who were considered to have long-term (chronic) infections had urogenital tract infections at multiple consecutive clinical examinations over a period of more than 3 months. In addition, after this initial decline in infection load after the first 3 months, there did not appear to be a significant trend in infection levels over time for koalas with long-term urogenital tract infections (Supplementary Fig. S2).

### Disease progression was not influenced by sex or age

There was no significant difference in the median ages of koalas with different infection outcomes (Kruskal-Wallis test, *p* = 0.766) (Supplementary Fig. S3). In addition, although 91% (10/11) of koalas that were able to resolve an infection were female, and only 9% (1/11) were male, the difference between the sexes in their ability to resolve an infection was not statistically significant (two-tailed Fisher’s exact test, *p* = 0.055).

### Only *omp*A genotypes E′ or G were detected at the ocular site, contrasting with urogenital tract infections

**Figure 3:**
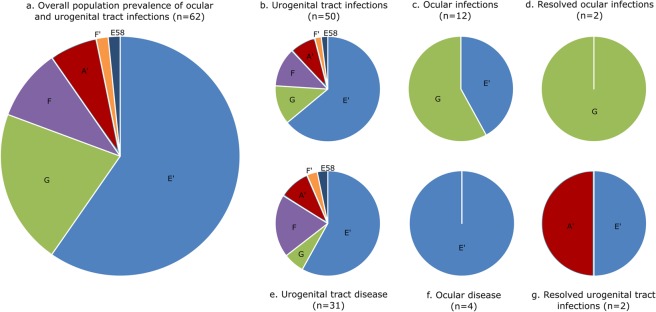
Prevalence of chlamydial strains, as characterised by *omp*A genotypes, relative to disease dynamics in a population of south-east Queensland koalas. E58 is identical to the livestock E58 *omp*A sequence.

From our longitudinal population study, we were able to resolve a total of 62, 359 nt *omp*A sequences from 72 ocular and urogenital tract samples taken from 47 koalas^6,12^. Genotyping of *C. pecorum* detected in the ocular swab samples showed that only *omp*A genotypes denoted E′ (42%; 5/12) or G (58%; 7/12) occurred at that site. In contrast, a variety of *omp*A genotypes, denoted A′, E′, F, F′, and G, and an *omp*A sequence identical to that of the livestock E58 strain, were all detected at the urogenital tract site (Fig. 3). Phylogenetic analysis of the 310 nt alignment from the VD 3–4 region of the *omp*A genotypes detected at both anatomical sites, and resolved in this study (n = 36), showed that they segregated into two major clades, with genotypes E′, F and F′ clustering together in one main clade (Supplementary Fig. S4).

Overall, *omp*A genotype E′ was the most prevalent genotype in all koalas, accounting for 60% (37/62) of all infections (Fig. 3a), and it was the most prevalent genotype detected at the urogenital tract site (64%; 32/50) (Fig. 3b). When present in multifocal infections (ocular and urogenital tract sites), genotype E′ was detected at both anatomical sites (n = 2) (Supplementary Table S2). The *omp*A genotype denoted G was also detected at the urogenital tract site (12%; 6/50), but it was much more common at the ocular site (58%; 7/12) (Fig. 3c). Unfortunately, although the *omp*A fragment was amplified in 29% (5/17) and 9% (5/55) of *C. pecorum* positive ocular and urogenital tract samples, respectively, we were not able to resolve these *omp*A sequences.

### Disease progression was associated with *omp*A genotypes F and F′ at the urogenital tract site and *omp*A genotype E′ at the ocular site

Koalas with *omp*A genotype F or F′ urogenital tract infections were significantly more likely to progress to disease at that anatomical site (100%; 7/7 vs 56%; 24/43) (two-tailed Fisher’s Exact Test, *p* = 0.035). There was also a higher relative prevalence of genotype F or F′ in urogenital tract infections that progressed to disease, in comparison to the overall population prevalence of genotype F or F′ (23%; 7/31 vs 11%; 7/62) (Fig. 3e vs 3a), but this difference was not statistically significant (two-tailed Fisher’s Exact Test, *p* = 0.218). All genotype F and F′ urogenital tract infections progressed to disease (n = 7), and genotype F was not detected in urogenital tract infections that were resolved by the host (n = 2). Interestingly, the median genotype F and F′ urogenital tract infection loads were a log higher than other genotypes (Supplementary Table S3), but this difference was not statistically significant (Kruskal-Wallis test, *p* = 0.142).

Koalas with *omp*A genotype E′ ocular infections were significantly more likely to progress to disease at that anatomical site (80%; 4/5 vs 0%; 0/7) (two-tailed Fisher’s Exact Test, *p* = 0.01). In all cases of ocular disease where an *omp*A genotype was determined (n = 4), genotype E′ infections were detected, and genotype E′ was not detected in ocular infections that were resolved by the host (n = 2) (Fig. 3d vs 3c). Interestingly, the median genotype E′ ocular infection load was a log higher than other genotypes (Supplementary Table S3), but this difference was not statistically significant (Kruskal-Wallis test, *p* = 0.121). In addition, although genotype G was the most prevalent genotype detected in ocular infections (n = 7), it was never associated with disease at that anatomical site.

### The *omp*A-characterised chlamydial strains in some long-term urogenital tract infections change detectable genotypes over time

**Figure 4:**
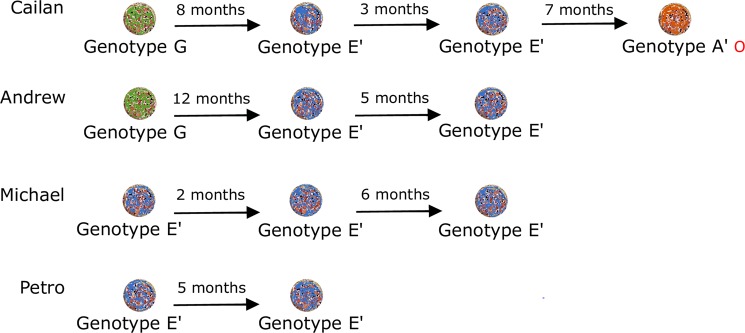
Chlamydial strain dynamics, as characterised by *omp*A genotypes (highlighted in colour), in long-term urogenital tract infections in individual koalas from a population of south-east Queensland koalas. Ο developed disease.

For koalas with long-term urogenital tract infections (more than 3 months), the *omp*A genotype detected at that anatomical site was genetically distinct over time in 50% (2/4) of cases (Fig. 4). For the remaining 50% (2/4) of cases, the *omp*A genotype detected at that anatomical site, genotype E′, was identical across the 5 to 8-month period. Genetically diverse infecting genotypes were not associated with an increase in infection load but were associated with disease progression in 25% (1/4) of koalas. The novel genotype associated with disease progression was the *omp*A genotype denoted A′. Unfortunately, due to the low number of cases, significant trends regarding strain-specific disease progression in koalas with long-term urogenital tract infections were not able to be determined.

### Unlike overall urogenital tract disease progression, infection load and *omp*A-characterised chlamydial strains did not appear to be important drivers of ‘severe’ chlamydial disease

During the longitudinal population study, 31% (158/503) of the total koalas in the population developed chlamydial disease at some point. Interestingly, 10% (16/158) of koalas who developed chlamydial disease had ‘severe’ disease, defined as disease that warranted euthanasia on welfare grounds due to a poor prognosis (n = 12) or that would have resulted in the death of the koala without veterinary intervention (n = 4). In total, 9% (9/98) of females with disease and 12% (7/60) of males with disease presented with ‘severe’ disease. All the koalas with ‘severe’ chlamydial disease had either cystitis, or cystitis and reproductive or ocular disease (i.e. multifocal disease).

Of those koalas with ‘severe’ chlamydial disease, where qPCR results were available, only 46% (5/11) had a detectable infection at the urogenital tract site at the time of their diagnosis of ‘severe’ chlamydial disease, despite all being diagnosed with cystitis (Supplementary Table S4). For koalas with a detectable infection at the urogenital tract site, the median infection load was 386 copies/µL (range 171 to 37,840 copies/µL), which was not significantly different from other koalas that developed disease (Mann-Whitney U = 28.5, *p* = 0.209) (Fig. 2d). Of those infected koalas with ‘severe’ chlamydial disease, where *omp*A genotyping was possible, 57% (4/7) had genotype E′ infections and 43% (3/7) had genotype F infections.

Where koalas were monitored for a period of time before developing ‘severe’ chlamydial disease, and qPCR results were available (n = 5), 100% had previously received antibiotic treatment for either urogenital tract infections (n = 2) or urogenital tract disease (n = 3). The median age of koalas with ‘severe’ chlamydial disease was 4.1 years (range 1.3 to 8 years), which was not significantly different from other koalas that developed disease (Mann-Whitney U = 216.5, *p* = 0.503).

### Prior chlamydial exposure was not a necessary driver of female reproductive tract disease progression

In total, 63% (62/98) of the diseased female koalas were diagnosed with reproductive tract disease. An additional 11 female koalas were suspected to have reproductive tract disease due to a failure to reproduce or subtle sonographic changes, but a definitive diagnosis was not achievable. Disease progression in the reproductive tract resulted from an infection acquired shortly after sexual maturity (after approximately 13 months of age) in 24% (15/62) of koalas with reproductive tract disease (Supplementary Table S5), indicating that prolonged or repeated exposure to chlamydial antigens was not a necessary driver. Where sexually mature koalas were monitored for a period of time before developing reproductive tract disease and qPCR results were available, 50% (3/6) had previous urogenital tract infections and 62% (8/13) were previously diagnosed with urogenital tract disease: either cystitis (n = 7) or cystitis and conjunctivitis (n = 1).

Only 53% (16/30) of koalas with clinically detectable reproductive tract disease and available qPCR results had a detectable infection at their urogenital tract site, when previously treated koalas were excluded. Two koalas who received surgical treatment for bilateral reproductive tract disease (but not antibiotics, due to a lack of available effective treatment at the time) continued to shed *Chlamydia* from their urogenital sinus for prolonged periods (more than 2 years in one case) (Supplementary Fig. S1). This demonstrates that surgical treatment alone does not achieve microbiological cure. For koalas with reproductive tract disease, only the *omp*A genotype denoted E′ (n = 3) was detected at the urogenital tract site. Unfortunately, due to the small sample size, significant trends regarding strain-specific reproductive tract disease progression in koalas were not able to be determined, and this finding may simply reflect the higher overall population prevalence of genotype E′ at the urogenital tract site.

### Successful treatment of chlamydial disease conferred less than 6 months of ‘protection’ against reinfection and subsequent disease progression

Of the koalas who were successfully treated (disease resolution and/or microbial cure at their post treatment examination) for urogenital tract disease with antibiotics (60 mg/kg chloramphenicol SC for 14 to 28 days)^21^, and qPCR results were available, 9 koalas subsequently re-presented with a newly acquired chlamydial infection and a new episode of chlamydial disease. There was a median of only 116 days (range 33 to 343 days) before a newly acquired infection was detected and a median of only 131 days (range 33 to 337 days) before a subsequent episode of chlamydial disease was detected. In 83% (5/6) of koalas who re-presented, where *omp*A genotyping was possible, genotype E′ infections were detected at the urogenital tract site at both clinical examinations, likely reflecting the higher overall population prevalence of this genotype at the urogenital tract site.

## Discussion

Some northern Australian koala populations are declining towards extinction due to threats that include habitat degradation and chlamydial disease^1–3^. To stabilise these populations, greater investment in population management strategies is necessary. The efficiency and success of such management strategies rely, in part, on a thorough understanding of chlamydial epidemiology and disease dynamics. Due to the paucity of comprehensive, longitudinal population studies^9,11^, largely resulting from historical limitations in the diagnosis of chlamydial infection and disease and the expense associated with such research, this knowledge is lacking. To address this knowledge gap, we utilised a unique data set from a wild koala population in SE Qld to reveal several new, major findings. Firstly, almost two thirds of the chlamydial infections detected and monitored in the population, progressed to chlamydial disease, challenging the notion that the majority of chronic chlamydial infections in koalas do not progress to clinical disease. Secondly, disease progression at the urogenital tract site was associated with the chlamydial infection load, and shedding from the urogenital tract was significantly higher in recently infected koalas. Thirdly, repeated or ongoing chlamydial exposure to did not appear to be necessary for negative sequelae to develop, such as infertility and mortality. Fourthly, some strains, as characterised by *omp*A genotyping, reflected possible tissue tropisms and pathogenicity, and reinfections with novel genotypes may have been responsible for some long-term infections. Finally, successful antimicrobial treatment provided only short-term protection against reinfection and subsequent disease progression in susceptible koalas.

In contrast to the current paradigm derived from opportunistic, cross-sectional studies^10,11,22,23^, when monitored longitudinally, we found that only 5% of chlamydial infections remained chronic and asymptomatic in this SE Qld koala population. Instead, our data show that 66% of chlamydial infections progressed to disease and the remaining 29% of infections were resolved by the koala between clinical examinations, without progressing to detectable disease. Further, in 67% of koalas with long-term chlamydial infections, we observed genetically distinct chlamydial strains, as characterised by their *omp*A genotypes, between clinical examinations. This suggests that koalas with long-term chlamydial infections may have been repeatedly resolving their infections before being reinfected with novel genotypes or shifting between different dominant genotypes in a mixed infection. Across a range of hosts, chlamydial infections have often been characterised by prolonged carriage in a latent form, without causing disease, or exerting only subclinical effects on the host^24,25^. However, even for the minority of koalas that had long-term, asymptomatic infections in our study, it is possible that subtle disease may have been overlooked during their clinical examinations due to limitations in the sensitivity of ultrasonography alone at detecting reproductive tract disease in both male and female koalas^26,27^. If the threat of chlamydial disease is to be successfully managed for koalas, these discoveries support a move away from the reliance on a clinical diagnosis for *Chlamydia* in both wildlife hospital and field settings and demonstrate the importance of access to point-of-care molecular diagnostics^10^. This will enable the identification of infections in a timely fashion, allowing veterinary interventions to avert disease progression and negative sequelae.

Determining the specific factors that drive chlamydial disease progression in susceptible individuals is an important priority in chlamydial research. We found that urogenital tract disease progression was significantly associated with the chlamydial infection load at that anatomical site, and that koalas with high infection loads at their urogenital tract site progressed to disease more rapidly. Further, our data show that infection loads at the urogenital tract site were significantly higher when koalas acquired an infection in the preceding three months, with loads then declining to a relatively stable level in chronically infected koalas. Shedding of *Chlamydia* is likely to be governed by the effectiveness of the host immune response and pathogen growth kinetics, and further focused study investigating the host immune response and levels of shedding during infection would be valuable. We also found that effective antibiotic treatment of chlamydial disease^21^ conferred only short-term immunity to reinfection, even by the same *omp*A-characterised chlamydial strain. Consistent with the theory that immune-mediated pathogenesis is a driver of the severity of chlamydial disease^16,28^, ‘severe’ chlamydial disease was associated with prior chlamydial exposure. Some koalas, however, developed negative sequelae such as infertility and mortality, without prior or ongoing exposure to *Chlamydia*, demonstrating that severe immune-mediated disease can also occur in naïve individuals^22^. Together, these findings highlight the fact that both new and repeated chlamydial infections can have serious consequences, and management interventions that reduce the transmission of *Chlamydia* and improve immune responses, such as vaccination, will be important in the future.

Several studies have suggested that genetically distinct chlamydial strains, as characterised by *omp*A genotypes, may reflect both tissue tropisms and pathogenicity in koalas^11,29^, demonstrating the complexity of chlamydial pathogenesis. Interestingly, only genotypes G and E′ were detected at the ocular site in our study, and the restricted distribution of genotypes at the ocular site relative to the urogenital tract site suggests there may be some genotype-specific tissue tropisms of *C. pecorum* in koalas. This is consistent with the findings of Phillips *et al*.^29^ in koalas and *C. trachomatis* research in humans^30^. Interestingly, our data also show that koalas infected with *omp*A genotype F and F′ strains at the urogenital tract site and genotype E′ strains at the ocular site were significantly more likely to progress to disease at their respective anatomical sites. In addition, these *omp*A-characterised strains resulted in infection loads that were a log higher than other genotypes, suggesting that they might be more pathogenic. This finding is consistent with hospital-based studies that identified strains denoted *omp*A genotype F in 7/8 diseased koalas^31^ and strains denoted *omp*A genotype F as the most widely distributed genotype in urogenital tract samples^23^. Further, Legione *et al*.^11^ reported higher chlamydial infection loads in Victorian koalas infected with genotype F strains at the urogenital tract site, but it was not clear whether those koalas were also diseased. Miyairi *et al*.^30^ linked the pathogenicity of strains of *C. trachomatis* to growth rates *in vitro*, postulating that these reflect the efficiency of host-pathogen interactions. Similar differences in *in vitro* growth rates have been reported for ocular and genital strains of koala *C. pecorum*^32^, and continuing investigation into strain-associated tissue tropisms and pathogenicity of *C. pecorum* in koalas is warranted.

A limitation of this study was that infecting chlamydial strains were characterised by genotyping using amplicon sequencing of the *omp*A gene. This method may have biased the findings towards infections containing a single population of genetically distinct *C. pecorum*, or more likely, towards the dominant genotype in infections containing mixed populations of genetically distinct *C. pecorum* strains. Culture-independent, probe-based genome capture and sequencing of clinical samples from koalas has shown that naturally occurring chlamydial infections frequently consist of mixed populations of genetically distinct *C. pecorum* strains^33^. In this study, amplified *omp*A sequences were not able to be resolved due to poor quality chromatograms in 29% and 9% of *C. pecorum* positive ocular and urogenital tract samples, respectively, and may indeed represent mixed infections. Whole genome sequencing could be employed to further characterise infecting chlamydial strains and assist in identifying mixed infections, however its application in large sample sizes is currently cost-prohibitive. As this method becomes more accessible in the future, it will improve our understanding of both the role of mixed genotype infections in chlamydial pathogenicity and tissue tropisms, and whether long-term infections with chlamydial strains that were genetically distinct over time were due to repeated resolution and reinfection or chlamydial strain dynamics of mixed infections.

In conclusion, most of the published literature on chlamydial epidemiology and disease dynamics in koalas originates from data sets that fail to accurately represent these interactions in wild koala populations. Instead, our current study utilised a comprehensive, longitudinal data set from a wild koala population in SE Qld to reveal several key findings. Our findings demonstrate the importance of point-of-care molecular diagnostics that enable the prompt detection of chlamydial infections, so that chlamydial disease progression can be prevented with veterinary interventions such as antimicrobial treatment or vaccination. These findings also support further investigation of vaccination programs that aim to reduce transmission of *Chlamydia* in at-risk populations and thereby improve population viability. Finally, we provide new focus for future chlamydial research across a range of hosts, including humans, to elucidate the host-associated factors that contribute to resistance to chlamydial disease progression and the chlamydial strains with increased pathogenicity and tissue tropisms.

## Methods

### Animals

A koala management program was conducted as part of a large-scale infrastructure project in SE Qld (27.0946°S, 152.9206°E) between 2013 and 2017. The program involved the capture, telemetric monitoring and veterinary management of 503 free-living independent and near-independent (starting to separate from their mother) koalas, an estimated 95% of the resident population^2^. Koalas were subject to thorough and standardised clinical examinations, conducted by koala-experienced veterinarians, at entry to and exit from the program and approximately every 6 months during the period of their monitoring (or more frequently if there were health or welfare concerns). At every clinical examination, aluminium-shafted, rayon-tipped swabs (Copan, Murrieta, California) were used to collect samples from the ocular conjunctiva and urogenital sinus (females) or urethra (males). Most veterinary procedures were conducted at the Endeavour Veterinary Ecology facilities in Toorbul, Queensland, to ensure consistency. Data were recorded in a purpose-designed database, using the FileMaker software (Apple, Sydney, NSW). A subset of the koalas in the koala management program was selected for inclusion in disease progression analyses (30 koalas) and chlamydial strain characterisation (47 koalas) in this study, based on the availability of longitudinal data sets and samples for analysis.

### Regulatory approvals

The koala management program was conducted under approvals issued by the Queensland Department of Agriculture and Fisheries (approvals CA 2012/03/597, CA 2013/09/719, CA 2014/06/777, CA 2015/03/852, and CA 2016/03/950). Work with koalas was authorised by scientific purposes permits issued by the Queensland Department of Environment and Heritage Protection (approvals WISP 11525212, WISP 16125415, WISP 13661313, WITK 14173714 and WISP 17273716). Swab samples were analysed under approval number AN/A/13/80 issued by the University of the Sunshine Coast Animal Ethics Committee. All experiments were performed in accordance with the relevant guidelines and regulations.

### Detection of chlamydial disease

Chlamydial disease diagnoses were based on the findings of clinical examinations, which included cytological examination of the urine sediment and sonographic examination of the urogenital tract (including kidneys), as previously described in Robbins *et al*.^21^.

### Classifying chlamydial disease

‘Severe’ chlamydial disease was defined as disease that warranted euthanasia on welfare grounds due to a poor prognosis (n = 12) or that would have resulted in the death of the koala without veterinary intervention (n = 4). At least two of the following criteria were necessary at the time of a diagnosis of disease for inclusion in the ‘severe’ disease category (if the koala was not euthanized): loss of more than 10% of body weight, dehydration, decline in body condition (2 scores on a 10-score system) and hypoproteinaemia. Two of the koalas in this group had all four criteria, one koala had two criteria with a urogenital tract prolapse and one koala had two criteria with renal pathology and metritis.

### Chlamydial disease treatment

The treatment of chlamydial disease in the study population has previously been reported by Robbins *et al*.^21^. Briefly, treatment with 60 mg/kg of chloramphenicol (Chloramphenicol-150, Ceva, Glenorie, New South Wales), administered daily by subcutaneous injection for 14–28 days, resulted in sufficient resolution of clinical signs for long-term disease resolution in over 94% of treated koalas.

### Detection of chlamydial infections with qPCR

Swab samples collected from the ocular conjunctiva and urogenital tract during clinical examinations were stored at −20 °C until processing. Swab samples were mixed with 500 µL of phosphate-buffered saline and DNA was extracted from a 200 µL aliquot using a QIAamp DNA mini kit (Qiagen, Chadstone, Victoria) according to the manufacturer’s instructions. The extracted samples were analysed using qPCR methods modified from Jelocnik *et al*.^34^ targeting a 209 base pair region of a *C. pecorum*-specific conserved gene *CpecG_0573*. Specifically, the qPCR assays were carried out in a total volume of 10 µL, consisting of 5 µL iTaq Universal SYBR Green Supermix (Bio-Rad, Gladesville, New South Wales), 1.5 µL DNA/RNA-free water, 0.5 µM forward and reverse primer, and 2.5 µL DNA template. Samples were run in duplicate, and positive and negative controls were included in all qPCR assays. A standard curve was generated for quantification using a known concentration of *C. pecorum* genomic DNA diluted to 10^7^–10^1^ copies/µL. Samples with less than 10 copies/µL were below the detectable limit of the assay and were reported as negative.

### *Chlamydiapecorumomp*A genotyping

A subset of 41 ocular and urogenital samples from *C. pecorum* qPCR positive koalas were genotyped by targeting a 359 base pair region of the V3 and V4 regions of the *omp*A gene, using methods modified from Nyari *et al*.^6^. Specifically, the conventional PCR assay was carried out in a total reaction volume of 50 µL, consisting of 25 µL of 2x HotStar Taq (Qiagen, Chadstone, Victoria), 20 µL DNA/RNA-free water, 0.3 µM forward and reverse primer, and 2 µL DNA template. Positive and negative controls were included in each assay. The PCR products were electrophoresed on a 1.5% agarose gel, followed by visual confirmation under a UV transilluminator, and 41 amplicons were bidirectionally sequenced in Macrogen Inc. (Korea).

Sequence analyses were performed in Geneious v.12^35^. The forward and reverse chromatograms were assessed for quality, and consensus sequences were obtained. A set of 36 sequences were chosen for further analysis, based on the criteria that both chromatograms were of a high quality. The 36 sequences were analysed using BLAST against the nr/nt nucleotide database (https://blast.ncbi.nlm.nih.gov/Blast.cgi) to evaluate their identity. The resulting top BLAST hit for the *omp*A genotype was recorded for each sequence. An additional 31 ocular and urogenital samples from *C. pecorum* qPCR positive koalas in the study population had previously been genotyped in studies by Nyari *et al*.^6^ and Waugh *et al*.^12^ and were included in our analyses. To evaluate phylogenetic relationships, an approximate maximum likelihood phylogenetic tree was constructed using the 310 nt MAFFT alignment of the 36 *C. pecorum omp*A gene fragment sequences with FastTree^36^, as implemented in Geneious v.12.

### Statistical analyses

Statistical analyses were performed using the Kruskal-Walis test, the Mann-Whitney U test or the two-tailed Fisher’s Exact test as appropriate (infection loads were not normally distributed). All statistical analyses were performed with the SPSS software package, and the statistical significance of all tests was concluded at *p-values* of ≤0.05.

## Supplementary information

Supplementary figures and tables
