# Supplementary material for: Longitudinal study of wild koalas (Phascolarctos cinereus) reveals chlamydial disease progression in two thirds of infected animals
Source: Sci Rep. 2019 Sep 13;9:13194. doi: 10.1038/s41598-019-49382-9 (PMC6744427; doi:10.1038/s41598-019-49382-9)
Supplement: Supplementary file 1 — Supplementary figures and tables [file 41598_2019_49382_MOESM1_ESM.pdf]

Longitudinal study of wild koalas (*Phascolarctos cinereus*) reveals chlamydial disease progression in two thirds of infected animals

Amy Robbins[1,2], Jonathan Hanger[2], Martina Jelocnik[1], Bonnie L Quigley[1], Peter Timms[1]*

S1 Table. Sampling schedule and infection outcomes for individual koalas from a population of south-east Queensland koalas.

NAME	SAMPLING 1	SAMPLING 2	SAMPLING 3	SAMPLING 4	SAMPLING 5	SAMPLING 6	SAMPLING 7	SAMPLING 8
Butter	10/04/2013	19/10/2013	8/04/2014	1/10/2014				
Josie	21/05/2014	20/11/2014	20/05/2015	11/11/2015				
Julia	4/04/2013	4/04/2014	15/10/2014					
Tamara O	6/11/2013	30/10/2014	28/04/2015					
Tamara O				26/10/2015	30/04/2014	21/04/2016		
Cora	9/01/2015	29/07/2015						
The Hoff	22/04/2013	3/07/2013	22/10/2013					
Doddy	25/03/2013	28/05/2013	17/09/2013					
Madison	10/10/2014	11/12/2014	15/04/2015	15/10/2015	14/06/2016	22/11/2016	3/02/2017	
Jadore	1/04/2014	30/09/2014						
Jadore			25/05/2015					
Karen	9/06/2013	8/10/2013	4/04/2014					
Karen				22/01/2015	19/02/2015	19/05/2015		
Bubbles	23/04/2013	3/07/2013	14/10/2013	15/04/2014				
Bubbles					9/01/2015	10/02/2015	22/04/2015	
Kapok	1/02/2014	25/06/2014	5/02/2015	4/03/2015	24/06/2015			
Petro	6/11/2013	30/04/2013	24/10/2014	16/04/2015	1/09/2015			
Cowboy	6/06/2013	5/12/2013	13/05/2014					
Susan	26/03/2013	8/10/2013	20/12/2013	18/03/2016	8/09/2016			
Susan						8/12/2016		
Gauthier	6/01/2014	5/05/2014	11/02/2015	7/07/2015	15/01/2016	25/10/2016		
Cailan	14/04/2014	16/10/2014	13/05/2015	10/06/2015	16/09/2015	4/04/2016	17/04/2016	8/09/2016
Walt	2/05/2014	18/09/2014	6/05/2015					
Izzy	13/08/2015	27/10/2015						
Copper	21/08/2014	30/12/2014						
Cougar	1/06/2013	12/03/2014						
Davey	15/09/2016	29/11/2016						
Ozone	13/09/2013	6/01/2014						
Tanja	17/03/2016	13/04/2016	17/01/2017					
Midori	25/05/2015	11/09/2015	8/10/2015					
Midori				10/11/2015	7/12/2015	20/12/2015		
Brodie	8/09/2015	5/11/2015	8/01/2016					
Brodie				4/02/2016	4/07/2016			
Anna	31/03/2015	1/10/2015	24/11/2015	21/12/2015	15/04/2016			
Andrew	11/10/2013	4/04/2014	18/12/2014	8/04/2015	30/09/2015	6/04/2016	19/09/2016	31/10/2016
Michael	12/08/2015	7/10/2015	16/12/2015	12/01/2016				
Michael					19/04/2016	16/06/2016	8/12/2016	
Patricia	18/06/2015	18/01/2016	9/05/2016	6/06/2016	27/10/2016			

Infection only | Infection and disease at the same time | Chronic infection then disease | Chronic infection and disease

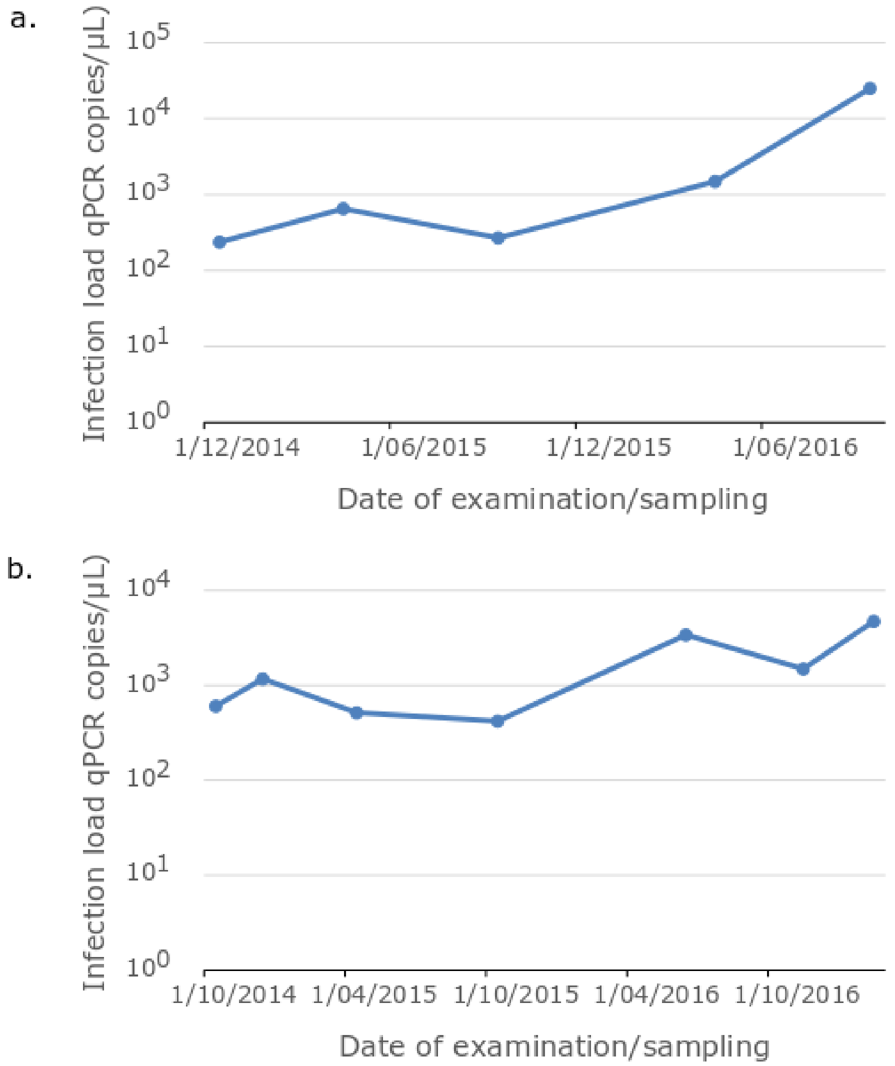

S1 Figure. Examples of long-term/chronic, asymptomatic urogenital tract infections in individual koalas from a population of south-east Queensland koalas.

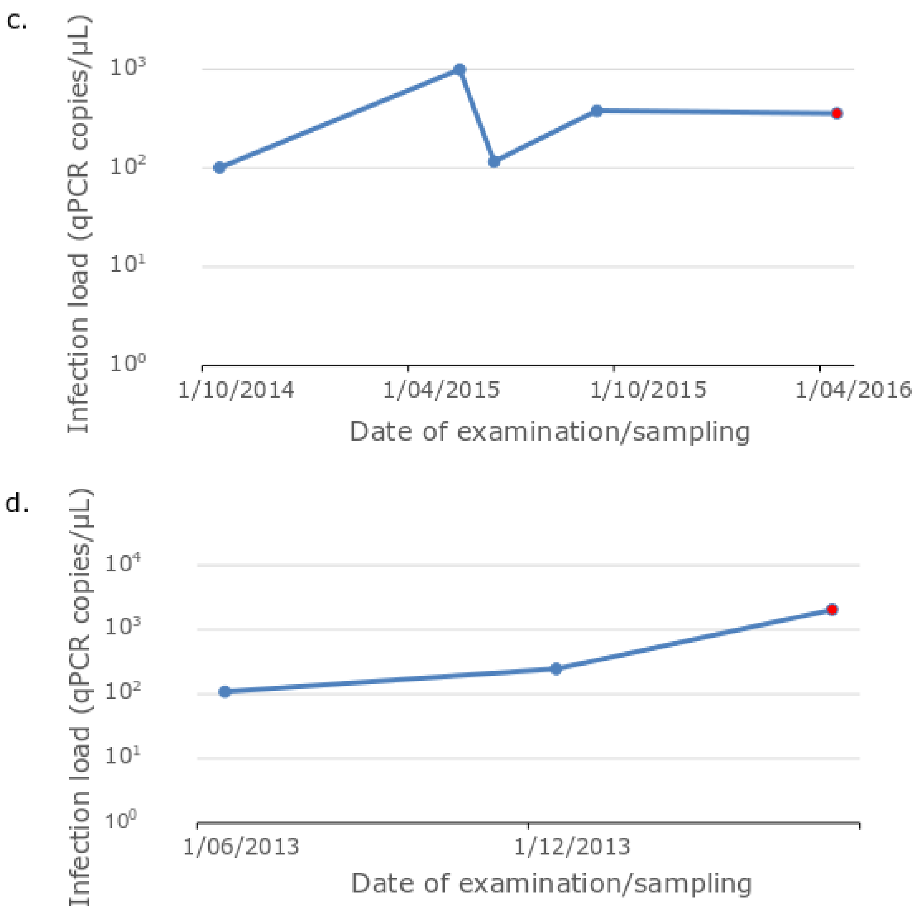

S2 Figure. Examples of chronic urogenital tract infections that progressed to disease in individual koalas from a population of south-east Queensland koalas. ● indicates disease.

S2 Table. Chlamydial strains, as characterised by *omp*A genotypes, in multifocal chlamydial infections in individual koalas from a population of south-east Queensland koalas.

Name	Ocular *omp* A genotype	Urogenital tract *omp* A genotype
Andrew	Could not be resolved	G
Beast	E'	E'
Cailan	Could not be resolved	G
Fury	E'	E'
Izzy	Could not be resolved	G
Tash	G	G

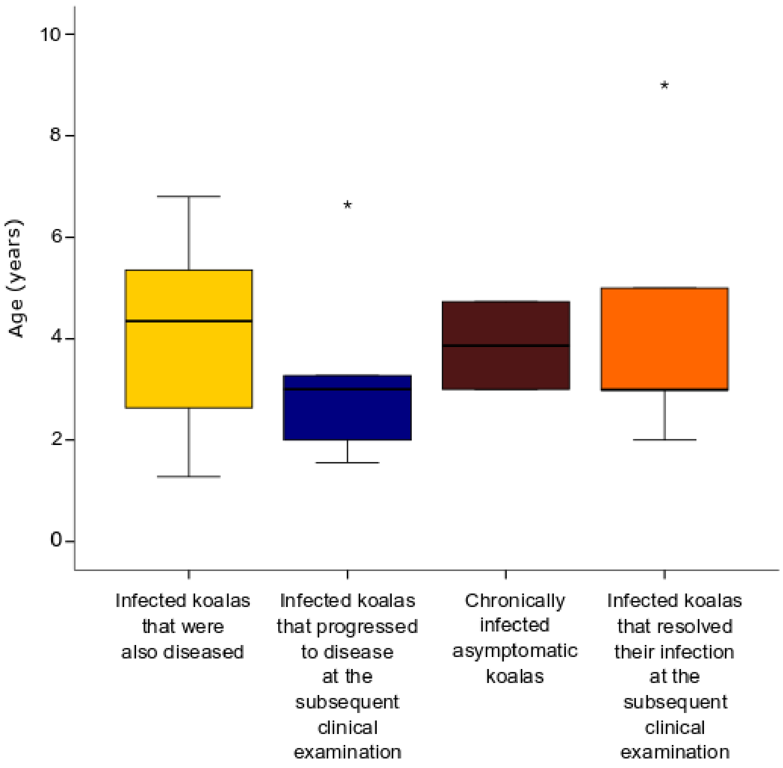

S3 Figure. Age distribution across different infection outcomes in a population of south-east Queensland koalas.

S3 Table. Median urogenital tract infection loads for each chlamydial strain, as characterised by *omp*A genotypes, in a population of south-east Queensland koalas.

Anatomical site	*omp* A genotype	Median qPCR load (copies/µL)	Range (copies/µL)
Urogenital tract	E'	3.69E+03	1.60E+01 - 1.37E+05
	G	1.93E+03	1.01E+02 - 1.76E+04
	F	3.53E+04	3.96E+03 - 1.34E+06
	A'	6.06E+02	3.14E+02 - 9.52E+02
	F'	2.09E+04	2.09E+04
	E58^	2.03E+03	2.03E+03
Ocular site	E'	6.73E+04	6.92E+02 - 1.34E+05
	G	4.70E+01	4.70E+01 - 5.20E+01

^ E58 is identical to the livestock E58 *omp*A sequence.

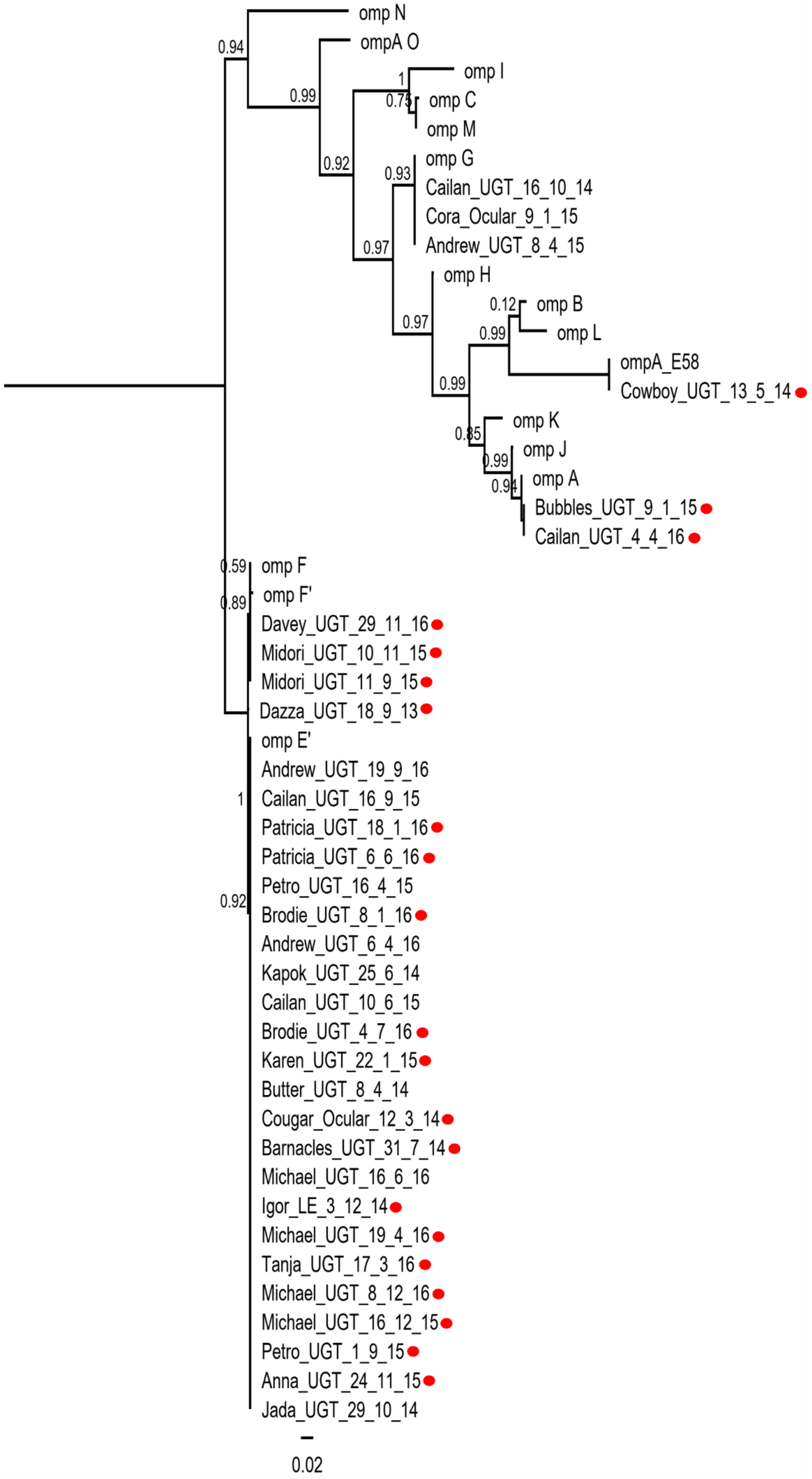

omp N
ompA O
omp I
omp C
omp M
omp G
Cailan_UGT_16_10_14
Cora_Ocular_9_1_15
Andrew_UGT_8_4_15
omp H
omp B
omp L
ompA_E58
Cowboy_UGT_13_5_14
omp K
omp J
omp A
Bubbles_UGT_9_1_15
Cailan_UGT_4_4_16
omp F
omp F'
Davey_UGT_29_11_16
Midori_UGT_10_11_15
Midori_UGT_11_9_15
Dazza_UGT_18_9_13
omp E'
Andrew_UGT_19_9_16
Cailan_UGT_16_9_15
Patricia_UGT_18_1_16
Patricia_UGT_6_6_16
Petro_UGT_16_4_15
Brodie_UGT_8_1_16
Andrew_UGT_6_4_16
Kapok_UGT_25_6_14
Cailan_UGT_10_6_15
Brodie_UGT_4_7_16
Karen_UGT_22_1_15
Butter_UGT_8_4_14
Cougar_Ocular_12_3_14
Barnacles_UGT_31_7_14
Michael_UGT_16_6_16
Igor_LE_3_12_14
Michael_UGT_19_4_16
Tanja_UGT_17_3_16
Michael_UGT_8_12_16
Michael_UGT_16_12_15
Petro_UGT_1_9_15
Anna_UGT_24_11_15
Jada_UGT_29_10_14
0.94
0.99
1
0.75
0.92
0.93
0.97
0.97
0.12
0.99
0.99
0.85
0.99
0.94
0.59
0.89
1
0.92
0.02

S4 Figure. Phylogenetic analysis of chlamydial strains in a population of south-east Queensland koalas, as characterised by *omp*A genotypes, from both ocular and urogenital tract infections. ● indicates disease.

S4 Table. Chlamydial strains, as characterised by *omp*A genotypes, and infection loads (copies/µL) in individual koalas from a population of south-east Queensland koalas with 'severe' chlamydial disease.

Koala	Sex	Age	Left ocular	Right ocular	Urogenital tract	*omp* A genotype
2-D	Female	3.25				
Barnacles	Male	5.09			1.71E+02	E'
Bessie	Female	1.27			4.66E+03^	F
Bones	Male	2	0.00E+00	0.00E+00	0.00E+00	F
Brodie	Male	2.64	0.00E+00	0.00E+00	2.49E+02	E'
Bubba	Male	6	0.00E+00	0.00E+00	0.00E+00	
CJ	Female	4.15				
Cougar	Female	7	1.34E+05	1.34E+05	N/A	E'*
Lee	Male	2.44	0.00E+00	0.00E+00	0.00E+00	
Liam	Male	3.65				
Margo	Female	8	0.00E+00	0.00E+00	0.00E+00	
Midori	Female	5.81	0.00E+00	0.00E+00	3.78E+04	F
Palmer	Female	1.8				
Patricia	Female	6.16	0.00E+00	0.00E+00	5.24E+02	E'
Ramses	Male	8	0.00E+00	0.00E+00	0.00E+00	
Venom	Female	4	0.00E+00	0.00E+00	0.00E+00	

*based on ocular genotype as no urogenital tract sample available. ^ previously reported by Waugh *et al.* [10]

S5 Table. Ages of female koalas from a population of south-east Queensland koalas with 'early' reproductive disease.

Name	Age (years)
Astra	1.5
Bessie	1.27
Charlotte	2.55
Chris	2
Cox	1.58
Even	2
Frangi	2
Hestia	1.43
Jud	1.24
Kapok	2.18
Kelly	2.22
Kyarna	2
Rosie	2.22
Savannah	2.56
Tanja	1.78
